# A Multi-Scale Edge-Preserving Decomposition and Fusion Framework for Multi-Polarization Passive Millimeter-Wave Imaging

**DOI:** 10.3390/s26113577

**Published:** 2026-06-04

**Authors:** Xinpeng Chen, Fei Hu, Dong Zhu, Jinlong Su, Bo Fang, Jingyu Tao

**Affiliations:** 1 School of Electronic Information and Communications, Huazhong University of Science and Technology, Wuhan 430074, China; chen_xinpeng_hust@163.com (X.C.); dzhu@hust.edu.cn (D.Z.); sujl@hust.edu.cn (J.S.); d202381095@hust.edu.cn (B.F.); jingyu_tao@hust.edu.cn (J.T.); 2 National Key Laboratory of Science and Technology on Multispectral Information Processing, Huazhong University of Science and Technology, Wuhan 430074, China

**Keywords:** PMMW imaging, multi-polarization fusion, personnel security inspection, object detection

## Abstract

**Highlights:**

**What are the main findings?**

- A hierarchical GWACF decomposition and layer-specific fusion strategy significantly enhance concealed object contrast in multi-polarization PMMW images while preserving edge integrity.
- Experimental results confirm superior detection accuracy and robustness over mainstream methods, demonstrating reliable concealed object imaging on the human body.

**What are the implications of the main findings?**

- The significant boost in concealed-object contrast and edge fidelity reduces both false alarms and missed detections, establishing multi-polarization PMMW imaging as a robust, privacy-safe solution for operational security screening.
- The proposed hierarchical decomposition and fusion framework provides a robust, generalizable strategy for fully exploiting multi-polarization information, establishing a new reference for processing polarimetric passive imagery in security and remote sensing applications.

**Abstract:**

Passive millimeter-wave (PMMW) imaging technology has become a highly promising technology that can protect privacy in human body security inspections. However, most existing methods rely on single-pixel and single-polarization processing mechanisms, which often lead to discrete false-alarm pixels or missed detections in practical applications. Although multi-polarization information can provide richer distinguishing features, the current methods typically depend on limited Stokes parameters or artificially designed polarization features, lacking a systematic framework to fully exploit the intrinsic potential of multi-polarization information. In this paper, we propose a novel multi-scale edge-preserving decomposition model, termed Gaussian and weighted average curvature filtering (GWACF), to hierarchically decompose a multi-polarization PMMW image into three structural layers: base structural (BS) layer, coarse structural (CS) layer, and fine structural (FS) layer. Furthermore, we also propose a fusion strategy in which a gradient-domain pulse-coupled neural network (PCNN) is employed to fuse the texture-rich CS and FS layers, while the energy attribute fusion method is applied to the BS layer where primary structure and background information play a dominant role. This method effectively leverages complementary polarimetric information without introducing artifacts or compromising edge sharpness. Experimental results demonstrate that the proposed method effectively enhances the brightness temperature (BT) contrast of concealed objects. Compared with existing mainstream methods, it exhibits notable advantages in both detection accuracy and robustness.

## 1. Introduction

Against the backdrop of an increasingly complex global security landscape and the public’s growing demand for safe travel, there is a pressing need for efficient, reliable, and privacy-respecting personal security screening technologies, which has become a key focus for both academia and industry [1,2,3]. Conventional metal detectors and handheld scanners are limited to identifying metallic objects, while being ineffective against non-metallic threats such as ceramic knives, plastic explosives, and liquid hazardous materials. Although active millimeter-wave imaging can penetrate clothing to reveal a human body outline [4,5], its use of transmitted electromagnetic waves has raised public concerns regarding potential health effects and personal privacy. Within this context, passive millimeter-wave (PMMW) imaging technology has gained prominence due to its distinct advantages. As a passive detection system, it only receives millimeter-wave signals radiated by the human body and concealed objects themselves, without the need for any form of electromagnetic wave emission [6,7]. Thus, it completely eliminates health risks and privacy violations at the physical level and is hailed as one of the ideal solutions for the next generation of human body security checks.

However, the practical application of PMMW imaging technology still faces a fundamental bottleneck: the low radiation brightness temperature (BT) contrast between the target and the background. Under indoor ambient temperature conditions, many non-metallic contraband objects exhibit radiation characteristics highly similar to those of human skin, resulting in extremely weak BT differences from the background in the final images, along with a low signal-to-noise ratio (SNR), which makes reliable detection and identification challenging [7,8]. Moreover, factors such as the material and thickness of clothing, fluctuations in environmental temperature, and the complex curvature of the human body further exacerbate image degradation [9,10] significantly limiting the detection performance and practical utility of traditional single-intensity imaging methods. To break through this bottleneck, researchers have turned their attention to another fundamental physical property of electromagnetic waves—polarization. Polarization information reveals the directional and structural characteristics of a target during the process of radiating or scattering electromagnetic waves, offering key insights that intensity images alone cannot provide [11]. Therefore, through polarimetric measurements, it becomes possible to extract material and structural “fingerprints” of concealed objects, enabling them to be more clearly distinguished from human skin amidst complex backgrounds.

Current techniques primarily rely on single-polarization images and single-pixel analysis modes. The information provided by a single polarization method is relatively limited, whereas multi-polarization techniques enable the acquisition of richer information [12,13]. Numerous studies have demonstrated the potential of multi-polarization sensing and processing across various applications, such as multi-polarization image enhancement [14,15], target segmentation [16,17], reflection interference suppression [18,19], material type identification [17,20], and wind direction/speed measurement [21,22]. Meanwhile, single-pixel processing methods often result in numerous discrete false positives or missed detections, thereby adversely affecting target detection and segmentation tasks. Consequently, many researchers advocate the adoption of multi-polarization imaging techniques to expand the dimensionality of BT data, enabling the extraction of richer scene information and enhanced detection performance. Previous studies have indicated that in-depth exploration and utilization of polarization information can significantly improve the detection capability of PMMW imaging [6,23]. To this end, parameters such as the degree of linear polarization (DoLP) [23], passive degree of polarization (PDoP) [11], linear polarization ratio (LPR) [16], and angle of polarization (AoP) [24] have been proposed as quantitative features for target detection in diverse scenarios, serving to measure the extent of target polarization. Such methods predominantly rely on polarization degree information for image segmentation to achieve target recognition. However, the polarization state is influenced not only by the target’s material composition but also by variations in target geometry and incidence angles, both of which can alter the observed polarimetric characteristics. These factors may lead to missed detections or false positives in polarization-based detection methods under specific conditions. Furthermore, the effective fusion of multi-dimensional polarization features with radiative intensity, spatial texture, and other relevant attributes is essential for constructing a robust detection system with low false alarm rates, representing a crucial step toward transitioning this technology from laboratory research to practical application.

To address the aforementioned challenges, this paper innovatively proposes a multi-polarization image fusion framework that combines multi-scale edge-preserving decomposition based on Gaussian and weighted average curvature filtering (GWACF) with a gradient-domain pulse-coupled neural network (PCNN). Unlike existing methods that mainly rely on handcrafted features such as Stokes parameters, simple polarization difference, or polarization ratio, our method, for the first time, cascades a weighted average curvature filter (WACF) with Gaussian filtering (GF) to hierarchically decompose an image into a base structural layer (BS), a coarse structural layer (CS), and a fine structural layer (FS). The uniqueness of this decomposition lies in the fact that WACF can simultaneously preserve edges and suppress noise, while the GF progressively strips away structural information at different scales, thus enabling each layer to carry complementary polarization features. Regarding the fusion strategy, we design distinct fusion rules for different layers: for the texture-rich CS and FS layers, a dual-channel PCNN modulated by multi-scale morphological gradient (MSMG) is adopted; for the BS layer that carries the dominant background, an energy-attribute weighted fusion scheme is used. This overall “decomposition–layering–adaptive fusion” pipeline has not been reported in the field of passive millimeter-wave (PMMW) multi-polarization image processing. In comparison, although the sub-region polarization fusion method [25] also exploits multi-polarization information, its fusion is primarily performed on sub-blocks of Stokes parameter images, lacking multi-scale separation of image structures. The Fisher vector polarization method [7] focuses on enhancing region classification through Fisher encoding, which essentially remains a feature-level post-processing rather than hierarchical fusion prior to image reconstruction. Deep learning-based methods [26] primarily rely on convolutional neural networks or Transformers for end-to-end object detection, and they have indeed achieved remarkable progress in performance. However, these methods typically require large amounts of annotated data and suffer from limited interpretability. In contrast, our method is a physics-driven image fusion method that does not rely on training data, thus offering better generalization and interpretability. Through edge-preserving decomposition and layer-wise differentiated fusion, it is physically more consistent with the millimeter-wave polarization scattering mechanism. The main contributions of this paper are:

(1) This paper designs a multi-scale edge-preserving decomposition model, which integrates Gaussian filtering with weighted average curvature filtering, termed GWACF. The model is designed to hierarchically represent multi-polarization PMMW images by decomposing them into three structural layers: the FS layer, the BS layer, and the CS layer. The FS layer and the CS layer jointly preserve the rich texture and specific structural information of the image, while the BS layer serves as the bottom-level expression of the image, capturing core elements such as primary structure and background content.
(2) In both the FS and CS layers, since they preserve the texture details and partial structural features of the image, the gradient-domain PCNN method performs well in fusing fine-textured images and is therefore employed for the fusion of these two layers. As for the BS layer, which contains the primary background information and overall structural contours of the image, the fusion effect at this level directly determines the overall quality of the final image. Given that the energy attribute (EA) fusion method performs exceptionally well when dealing with image with asymmetric information distribution, and is particularly suitable for coarse approximate image layers. Consequently, it is applied to the fusion of the BS layer.
(3) The proposed method was validated through multi-polarization PMMW imaging detection experiments. Experimental results demonstrate that the fusion method produces high-quality and robust imaging of concealed objects on the human body. Qualitative and quantitative comparisons with several competitive baseline methods further highlight its performance advantages.

The remainder of this paper is organized as follows. Section 2 presents the necessary preliminaries. The proposed fusion method is described in Section 3. Experimental results and performance analysis are provided in Section 4 to validate the effectiveness of our method. Finally, conclusions are given in Section 5.

## 2. Preliminaries

### 2.1. Analysis of the PMMW Imaging Model

The PMMW imaging security system detects concealed dangerous objects under a person’s clothing by measuring the effective radiation temperature distribution within the target scene. In practical experimental tests, the PMMW radiation temperature data received by the imaging system is the combined effect of the testing environment, the human body, and the target under examination.

**Figure 1:**
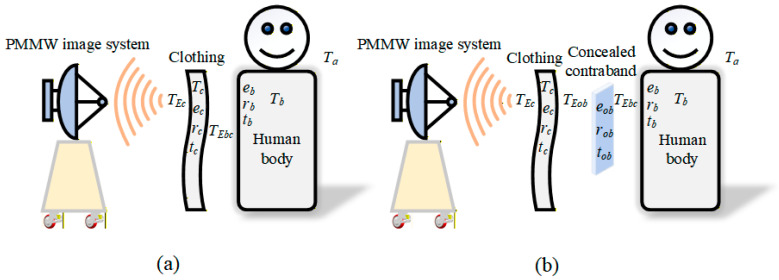
Schematic of the human body under radiation measurement. (**a**) The situation in which there are no concealed objects; (**b**) The situation in which objects are hidden beneath clothing.

As shown in Figure 1a, the radiation model for the case where no concealed objects are carried on the human body can be derived as follows:

(1) $$T_{Ec}=e_{c}T_{c}+r_{c}T_{a}+t_{c}T_{Ebc}$$

(2) $$T_{Ebc}=e_{b}T_{b}+r_{b}T_{Ec}+t_{b}T_{a}$$

where *T_Ec_* denotes the effective radiation temperature in front of the clothing, *T_c_* is the absolute temperature of the clothing, and *T_a_* represents the absolute temperature of free space; *e_c_*, *r_c_*, and *t_c_* represent the emissivity, reflectivity, and transmissivity of the clothing, respectively. *T_Ebc_* denotes the effective radiation temperature between the skin and the clothing, while *T_b_* is the absolute temperature of the human body; *e_b_*, *r_b_*, and *t_b_* represent the emissivity, reflectivity, and transmissivity of the human body, respectively.

By substituting Equation (2) into Equation (1), the effective radiation temperature obtained by the PMMW imaging system for the front clothing area of the human body can be expressed by the following expression:

(3) $$T_{Ec}=\frac{e_{c}T_{c}+r_{c}T_{a}+t_{c}(e_{b}T_{b}+t_{b}T_{a})}{1-t_{c}r_{b}}$$

In the scenario of a concealed object carried on the human body, as illustrated in Figure 1b, the radiation model is revised to the following:

(4) $$T_{Ec}=e_{c}T_{c}+r_{c}T_{a}+t_{c}T_{Eob}$$

(5) $$T_{Eob}=e_{ob}T_{ob}+r_{ob}T_{Ec}+t_{ob}T_{Ebc}$$

(6) $$T_{Ebc}=e_{b}T_{b}+r_{b}T_{Eob}+t_{b}T_{a}$$

where *T_Eob_* is the effective radiation temperature in front of the concealed object. *E_ob_*, *r_ob_*, and *t_ob_* represent the emissivity, reflectivity, and transmissivity of the concealed object, respectively. *T_ob_* denotes the absolute temperature of the concealed object.

Furthermore, substituting Equations (5) and (6) into Equation (4), Equation (3) can be rewritten as:

(7) $$T_{Ec}=\frac{\left(e_{c}T_{c}+r_{c}T_{a}\right)\left(1-T_{ob}r_{b}\right)+t_{c}\left[e_{ob}T_{ob}+t_{ob}\left(e_{b}T_{b}+t_{b}T_{a}\right)\right]}{1+t_{c}r_{ob}-t_{ob}r_{b}}$$

### 2.2. Pulse-Coupled Neural Network

The pulse-coupled neural network (PCNN), as a third-generation artificial neural network, offers the key advantage of requiring no parameter training. Decades of development have demonstrated its significant potential in various fields, including image denoising [27,28], image segmentation [29,30], target detection [31,32], and image fusion [33,34].

**Figure 2:**
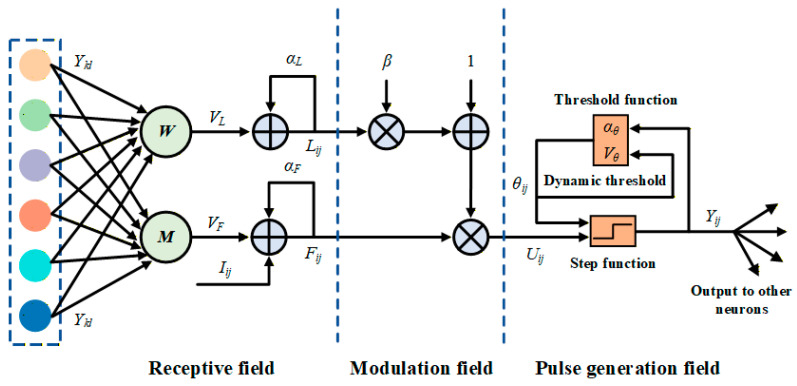
Schematic diagram of the PCNN neuron model.

A PCNN neuron (Figure 2) consists of three components: the receptive, modulation, and pulse generation fields, responsible for signal reception, internal state computation, and output, respectively. Its internal state computation is divided into two parts: connection and feedback inputs. The neuron communicates with its neighboring neurons via synaptic weights *M* and *W*, while receiving external input stimuli *I*. Each neuron retains its previous state, which decays over time.

(8) $$F_{ij}(n)=e^{-\alpha_{F}}F_{ij}(n-1)+I_{ij}+V_{F}\sum_{kl}M_{ijkl}Y_{kl}(n-1)$$

(9) $$L_{ij}(n)=e^{-\alpha_{L}}L_{ij}(n-1)+V_{L}\sum_{kl}W_{ijkl}Y_{kl}(n-1)$$

where *F_ij_* denotes the feedforward component of the neuron embedded at position (*i*, *j*) in the two-dimensional neural array, *n* represents the iteration number of the neuron, and *Y_kl_* corresponds to the neuronal output at time (*n* − 1). *L_ij_* refers to the corresponding connection input. All three components possess memory capabilities, with their state values decaying over time. The parameters *α_F_* and *α_L_* are the temporal decay constants for the feeding and connection inputs, respectively, governing their decay rates. The terms *V_F_* and *V_L_* denote the amplification coefficients of the connection weights for the feeding and connection input. These coefficients help to prevent the saturation of neuronal output, which is usually normalied to constants.

By coupling *F_ij_* and *L_ij_*, the internal activity *U_ij_* of the neuron is constituted, where *β* denotes the coupling strength. The mathematical expression for the internal activity is given by:

(10) $$U_{ij}(n)=F_{ij}(n)(1+\beta L_{ij}(n))$$

The output neuron *Y_ij_* is generated by comparing the internal activity *U_ij_* of the neuron with its dynamic threshold *θ_ij_*.

(11) $$Y_{ij}(n)=\left\{\begin{array}{l} 1,{\;U}_{ij}(n)>\theta_{ij}(n)\\ 0,\;\mathrm{otherwise} \end{array}\right.$$

The dynamic threshold *θ_ij_* is a variable parameter, as it sharply increases when the neuron fires (*Y_ij_* > *θ_ij_*). It then gradually decays back until the neuron fires again. This process is mathematically described by Equation (12), as shown below:

(12) $$\theta_{ij}(n)=e^{-\alpha_{\theta }}\theta_{ij}(n-1)+V_{\theta }Y_{ij}(n)$$

where *α_θ_* is the time constant of decay for the dynamic threshold *θ_ij_*, and *V_θ_* denotes the amplification factor associated with it.

## 3. Methodology

**Figure 3:**
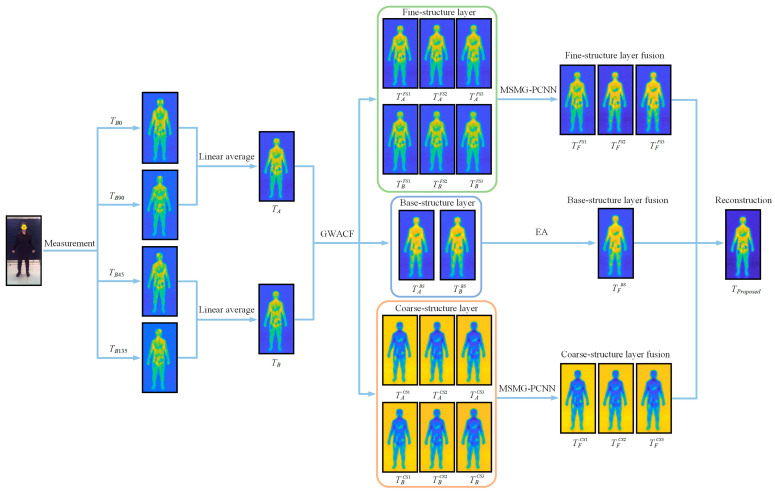
The framework of the proposed fusion strategy.

This section systematically presents the proposed multi-polarization PMMW image fusion method, whose overall workflow is depicted in Figure 3. The framework sequentially comprises the following five core modules: GWACF-based multi-scale decomposition, fusion of fine structural (FS) layer, fusion of coarse structural (CS) layer, fusion of base structural (BS) layer, and final image reconstruction.

The process begins by acquiring observational data at four linear polarization angles (0°, 45°, 90°, and 135°), which are recorded as *T_B_*_0_, *T_B_*_45_, *T_B_*_90_, and *T_B_*_135_, respectively. These polarization measurements are then linearly averaged to yield two components, *T_A_* and *T_B_*, defined as follows.

(13) $$T_{A}=\frac{T_{B0}+T_{B90}}{2}$$

(14) $$T_{B}=\frac{T_{B45}+T_{B135}}{2}$$

Subsequently, the GWACF decomposition algorithm is employed to decompose the *T_A_* and *T_B_* images into three distinct layers: the FS layer, CS layer, and BS layer. The corresponding BS layers are coalesced using an energy property-based fusion strategy. In contrast, the fusion of the FS and CS layers is achieved using a gradient-domain PCNN approach. The final fused image is reconstructed by integrating these combined layers.

From the perspective of physical rationality, the pairing strategy adopted in this paper—(*T_B_*_0_ and *T_B_*_90_) as one group, and (*T_B_*_45_ and *T_B_*_135_) as the other—exhibits clear completeness in the polarization basis. Each group forms an orthogonal polarization basis, enabling comprehensive characterization of any linear polarization state. These two groups are sensitive to the structural and dielectric properties of the target along the horizontal/vertical and diagonal directions, respectively, thereby providing complementary polarization signatures. Moreover, this pairing effectively suppresses the strong dependence of a single polarization channel on target orientation, significantly enhancing the robustness of concealed target detection while reducing redundancy and noise interference under the premise of preserving complementary polarization information.

As for the direct fusion of all four polarization channels, although theoretically feasible, it suffers from notable limitations. First, there exists informational redundancy among the four polarization angles; directly fusing them introduces substantial repetitive information into the fusion network, which may lead to feature competition and unstable fusion decisions. Second, simultaneously feeding all four polarization channels into the fusion model greatly increases computational complexity and may introduce nonlinear inter-channel interference, resulting in artifacts or edge blurring in the fused image.

In contrast, the proposed method first constructs two complementary components, *T_A_* and *T_B_*, which physically mitigate signal fluctuations caused by target orientation while preserving polarization anisotropy information. This provides a more robust input for subsequent edge-preserving decomposition and hierarchical fusion. Therefore, direct fusion of all four channels is not considered in this paper.

### 3.1. GWACF Multi-Scale Decomposition

For a 2D image *T*, its weighted average curvature filtering (WACF) [35] can be expressed as:

(15) $$\begin{array}{cc} G^{w}\left(T\right) & =k{\left\|\nabla T\right\|}_{2}G\left(T\right)\\ & ={\left\|\nabla T\right\|}_{2}\left(\nabla \cdot \frac{\nabla T}{{\left\|\nabla T\right\|}_{2}}\right) \end{array}$$

where ∇· and ∇ denote the divergence operator and the gradient operator, respectively.

In the case of *k* = 2, the expression of Equation (15) simplifies to:

(16) $$G^{w}\left(T\right)=\Delta T-\frac{T_{x}^{2}T_{xx}+2T_{x}T_{y}T_{xy}+T_{y}^{2}T_{yy}}{T_{x}^{2}+T_{y}^{2}}$$

where Δ is the isotropic Laplacian operator. *T_x_* and *T_y_* are the first-order partial derivatives along the *x* and *y* directions, respectively. *T_xx_*, *T_xy_* and *T_yy_* represent the corresponding second-order partial derivatives, respectively.

Within a 3 × 3 window, the investigation considers eight possible normalized half-window directions, with corresponding kernels generated for all eight cases:

(17) $$\begin{array}{l} f_{1}=\left[\begin{array}{ccc} 0 & 0 & 0\\ 1/6 & -1 & 1/6\\ 1/6 & 1/3 & 1/6 \end{array}\right],{\;f}_{2}=\left[\begin{array}{ccc} 0 & 1/6 & 1/6\\ 0 & -1 & 1/3\\ 0 & 1/6 & 1/6 \end{array}\right],\\ f_{3}=\left[\begin{array}{ccc} 1/6 & 1/3 & 1/6\\ 1/6 & -1 & 1/6\\ 0 & 0 & 0 \end{array}\right],{\;f}_{4}=\left[\begin{array}{ccc} 1/6 & 1/6 & 0\\ 1/3 & -1 & 0\\ 1/6 & 1/6 & 0 \end{array}\right],\\ f_{5}=\left[\begin{array}{ccc} 1/12 & 0 & 0\\ 1/3 & -1 & 0\\ 1/6 & 1/3 & 1/12 \end{array}\right],{\;f}_{6}=\left[\begin{array}{ccc} 0 & 0 & 1/12\\ 0 & -1 & 1/3\\ 1/12 & 1/3 & 1/6 \end{array}\right],\\ f_{7}=\left[\begin{array}{ccc} 1/12 & 1/3 & 1/6\\ 0 & -1 & 1/3\\ 0 & 0 & 1/12 \end{array}\right],{\;f}_{8}=\left[\begin{array}{ccc} 1/6 & 1/3 & 1/12\\ 1/3 & -1 & 0\\ 1/12 & 0 & 0 \end{array}\right]. \end{array}$$

Furthermore, the eight distance values *r_i_* can be computed using the eight kernels, respectively.

(18) $$r_{i}=f_{i}*T,\;i=1,2,\;\cdots ,8$$

where * denotes the convolution operation. The discrete form of Equation (16) can be expressed as:

(19) $$G^{w}\approx r_{h},\;h=\mathrm{argmin}\left\{\left|r_{i}\right|;\;i=1,2,\;\cdots ,8\right\}$$

For the purpose of analysis, the WACF procedure is represented as:

(20) $$T_{out}=\mathrm{WACF}\left(T_{in}\right)$$

where *T_out_* and *T_in_* represent the filtered output image and the original input image before filtering, respectively. WACF(·) denotes the WACF operation.

**Figure 4:**
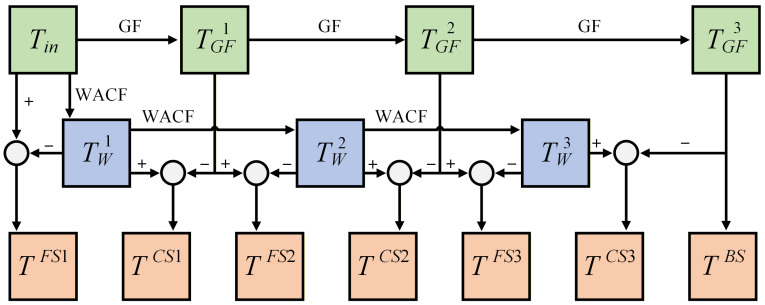
Flowchart of the GWACF decomposition method.

Based on this, the paper proposed an image decomposition method that integrates Gaussian filtering with weighted average curvature filtering, namely the GWACF method, whose overall workflow is illustrated in Figure 4. *T_in_* refers to the input image, $T_{GF}^{m}$ (*m* = 1, 2, 3) denotes the resultant image after the *m*-th Gaussian filtering operation, and $T_{W}^{m}$ is the result after performing the WACF operation. The decomposed layers $T^{\mathrm{FSm}}$, $T^{\mathrm{BS}}$ and $T^{\mathrm{CSm}}$ are then given by the following equations:

(21) $$T^{FSm}=\left\{\begin{array}{l} T_{in}-T_{W}^{m},\;m=1\\ T_{GF}^{m-1}-T_{W}^{m},\;m=2,3 \end{array}\right.$$

(22) $$T^{BS}=T_{GF}^{3}$$

(23) $$T^{CSm}=T_{W}^{m}-T_{GF}^{m}$$

where $T_{GF}^{m}$ and $T_{W}^{m}$ can be respectively calculated as follows:

(24) $$T_{GF}^{m}=\left\{\begin{array}{l} \mathrm{G F}\left(T_{in}\right),\;m=1\\ \mathrm{G F}\left(T_{GF}^{m-1}\right),\;m=2,3 \end{array}\right.$$

(25) $$T_{W}^{m}=\left\{\begin{array}{l} \mathrm{W A C F}\left(T_{in}\right),\;m=1\\ \mathrm{W A C F}\left(T_{\mathrm{G F}}^{m-1}\right),\;m=2,3 \end{array}\right.$$

where the symbol GF(·) denotes the Gaussian filtering operator.

After decomposition via the GWACF operator, *T_in_* can be composed of three distinct hierarchical levels.

(26) $$T_{\mathrm{in}}=\sum_{m=1}^{3}\left(T^{FSm}+T^{CSm}\right)+T^{BS}$$

Therefore, *T_A_* and *T_B_* in Figure 3 can be decomposed into $T_{A}^{FSm}$, $T_{B}^{FSm}$, $T_{A}^{BS}$, $T_{B}^{BS}$, $T_{A}^{CSm}$ and $T_{B}^{CSm}$ through Equations (21) to (23), respectively.

### 3.2. Fusion Strategy for Fine and Coarse Structural Layers

The multi-scale morphological gradient (MSMG) is an edge extraction method that integrates multi-scale strategies with mathematical morphological operations. By performing morphological operations at different structural element scales and fusing the results, it effectively enhances and captures image contours and detailed features with improved accuracy. The specific steps are as follows:

(1) The multi-scale structural elements are denoted as:

(27) $$MS_{v}=\underset{v}{\underbrace{MS_{1}⊕MS_{1}\cdots ⊕MS_{1}}},\;v=1,2,\cdots ,M$$

where *MS*_1_ denotes a basic structural unit, using a 3 × 3 matrix structuring element as the basic unit, i.e., *MS*_1_ = [1, 1, 1; 1, 1, 1; 1, 1, 1]. *v* represents the scale factor, and ⨁ indicates the morphological dilation operation.

(2) For an image *T*, its gradient feature *G_v_* is characterized by the morphological gradient operator as follows:

(28) $$G_{v}\left(T\right)=T⊕MS_{v}-T\Theta MS_{v}$$

where Θ denotes the morphological erosion operation.

(3) The output value *ρ* of MSMG can be computed as the weighted sum of the gradients across different scales:

(29) $$\rho =\sum_{v=1}^{N}w_{v}\cdot G_{v}\left(T\right)$$

where *w_v_* denotes the gradient weight at the *v*-th scale and can be expressed as:

(30) $$w_{v}=\frac{1}{2v+1}$$

**Figure 5:**
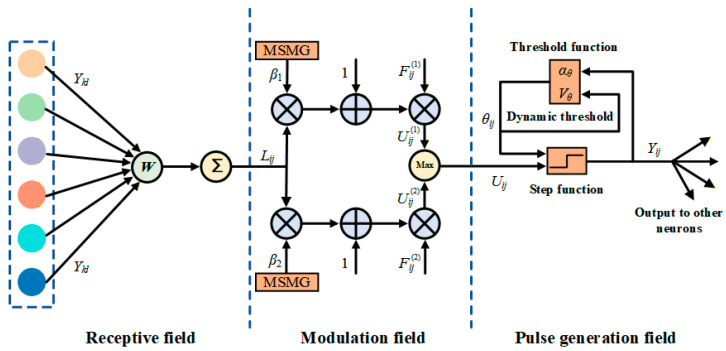
Gradient domain PCNN model with MSMG modulation.

Both the CS and FS layers carry the texture properties and partial structural characteristics of polarized images. The gradient-domain PCNN fusion strategy is well-suited for fusing images containing small-scale texture features, and is therefore adopted in this paper. Specifically, the CS and FS layers obtained by applying MSMG calculations to *T_A_* and *T_B_* are utilized as the connection strengths input in the PCNN, thereby constituting an MSMG-modulated dual-channel PCNN model, as shown in Figure 5. Its mathematical expression is given as follows.

According to the introduction in reference [36], under the premise of preserving the biological characteristics of the original model, the receptive field of the PCNN model can be simplified as:

(31) $$F_{ij}^{\left(1\right)}(n)=I_{ij}^{\left(1\right)}(n)$$

(32) $$F_{ij}^{\left(2\right)}(n)=I_{ij}^{\left(2\right)}(n)$$

(33) $$L_{ij}(n)=V_{L}\sum_{kl}W_{ijkl}Y_{kl}(n-1)$$

where $I_{ij}^{\left(1\right)}$ and $I_{ij}^{\left(2\right)}$ denote the input stimuli received by channel 1 and channel 2, respectively, with their magnitudes corresponding to the pixel values at location (*i*, *j*) in the two input images. *L_ij_* represents the connection parameter, and the connection weight amplification factor *V_L_* is set to 1. *W_ijkl_* denotes the connection weight matrix between neurons. When each neuron is positioned at the center of a 3 × 3 (or 5 × 5) weight matrix, its adjacent pixels correspond to the neurons within this matrix. The connection weights between neurons are closely related to their spatial distance.

Therefore, we define the connection weight as the inverse square of the Euclidean distance between connected neurons—specifically, the connection weight between neuron *ij* and neuron *kl* is given by:

(34) $$W_{\mathrm{ijkl}}=\left\{\begin{array}{cc} 0, & \mathrm{if}\;i=k=j=l\\ \frac{1}{\sqrt{{\left(i-k\right)}^{2}+{\left(j-l\right)}^{2}}}, & \mathrm{otherwise} \end{array}\right.$$

The modulation field is defined as:

(35) $$U_{\mathrm{ij}}(n)=\mathrm{Max}\left\{U_{ij}^{\left(1\right)}(n),{\;U}_{ij}^{\left(2\right)}(n)\right\}$$

where *U_ij_* denotes the internal activity of the dual-channel output, while $U_{ij}^{\left(1\right)}$ and $U_{ij}^{\left(2\right)}$ correspond to the internal activities of channel 1 and channel 2, respectively, which can be expressed as:

(36) $$U_{ij}^{\left(1\right)}(n)=F_{ij}^{\left(1\right)}(n)\left(1+\beta_{1}L_{ij}(n)\right)$$

(37) $$U_{ij}^{\left(2\right)}(n)=F_{ij}^{\left(2\right)}(n)(1+\beta_{2}L_{ij}(n))$$

Here, *β*_1_ and *β*_2_ represent the connection strengths of channel 1 and channel 2, respectively. As for the pulse generator field of this network, it can be represented by Equations (11) and (12).

Despite the notable advances PCNN has achieved in image fusion techniques, this method still exhibits a critical limitation: each pixel in its network architecture corresponds to an independent neuron. If PCNN is directly applied for fusion, pixels representing the same content within an image block may be activated asynchronously, leading to biased fusion decisions. Consequently, the final fused image may exhibit undesirable pixel-level mutations and block artifacts.

As mentioned above, the MSMG exhibits excellent capability in extracting image edge features. Therefore, it is employed as a pre-modulation processing unit for PCNN to effectively enhance spatial correlations across different layers. In this paper, the image results processed by MSMG are used as the connection strength input of the network, and the detailed mathematical representation is given as follows:

(38) $$\beta_{1}=\rho_{1}$$

(39) $$\beta_{2}=\rho_{2}$$

where *ρ*_1_ and *ρ*_2_ are defined as the outputs of the two input images under the MSMG operator, which can be derived from Equation (29).

Then, the fused FS and CS layers are denoted as:

(40) $$T_{F}^{FSm}(i,j)=\left\{\begin{array}{l} T_{A}^{FSm}(i,j),\;\mathrm{if}{\;U}_{ij}^{\left(1\right)}\geq U_{ij}^{\left(2\right)}\\ T_{B}^{FSm}(i,j),\;\mathrm{otherwise} \end{array}\right.$$

(41) $$T_{F}^{CSm}(i,j)=\left\{\begin{array}{l} T_{A}^{CSm}(i,j),\;\mathrm{if}{\;U}_{ij}^{\left(1\right)}\geq U_{ij}^{\left(2\right)}\\ T_{B}^{CSm}(i,j),\;\mathrm{otherwise} \end{array}\right.$$

where $U_{ij}^{\left(1\right)}$ and $U_{ij}^{\left(2\right)}$ can be obtained from Equations (36) and (37).

### 3.3. Fusion Strategy for Base Structural Layer

The BS contains the primary information of the polarized image (such as the main texture and the background). Given the information asymmetry inherent in polarization images, an energy-attribute fusion strategy is employed at this level to effectively balance disparities and achieve information complementarity and enhancement. The implementation of this strategy involves three main steps:

(1) Calculate the eigenvalues *H_A_* and *H_B_* of the BS layer, which are respectively expressed as:

(42) $$H_{A}={\bar{X}}_{A}+{\tilde{X}}_{A}$$

(43) $$H_{B}={\bar{X}}_{B}+{\tilde{X}}_{B}$$

where *H_A_* and *H_B_* represent the feature values of the BS layer. ${\bar{X}}_{A}$ and ${\bar{X}}_{B}$ represent the mean values of $T_{A}^{BS}$ and $T_{B}^{BS}$, respectively, while ${\tilde{X}}_{A}$ and ${\tilde{X}}_{B}$ denote the median values of $T_{A}^{BS}$ and $T_{B}^{BS}$, respectively.

(2) Calculate the energy attribute functions *E_A_* and *E_B_*, which are respectively given by:

(44) $$E_{A}(i,j)=e^{\left(\alpha_{G}\left|T_{A}^{BS}(i,j)-H_{A}\right|\right)}$$

(45) $$E_{B}(i,j)=e^{\left(\alpha_{G}\left|T_{B}^{BS}(i,j)-H_{B}\right|\right)}$$

where *E_A_* and *E_B_* represent the energy attribute functions of the BS layer. *α_G_* denotes the gain coefficient, we set *α_G_* = 4 in this paper.

(3) Obtain the final fused result $T_{F}^{BS}$ for the BS layer by weighted averaging:

(46) $$T_{F}^{BS}(i,j)=\frac{E_{A}(i,j)\cdot T_{A}^{BS}(i,j)+E_{B}(i,j)\cdot T_{B}^{BS}(i,j)}{E_{A}(i,j)+E_{B}(i,j)}$$

## 4. Validation Experiments

**Figure 6:**
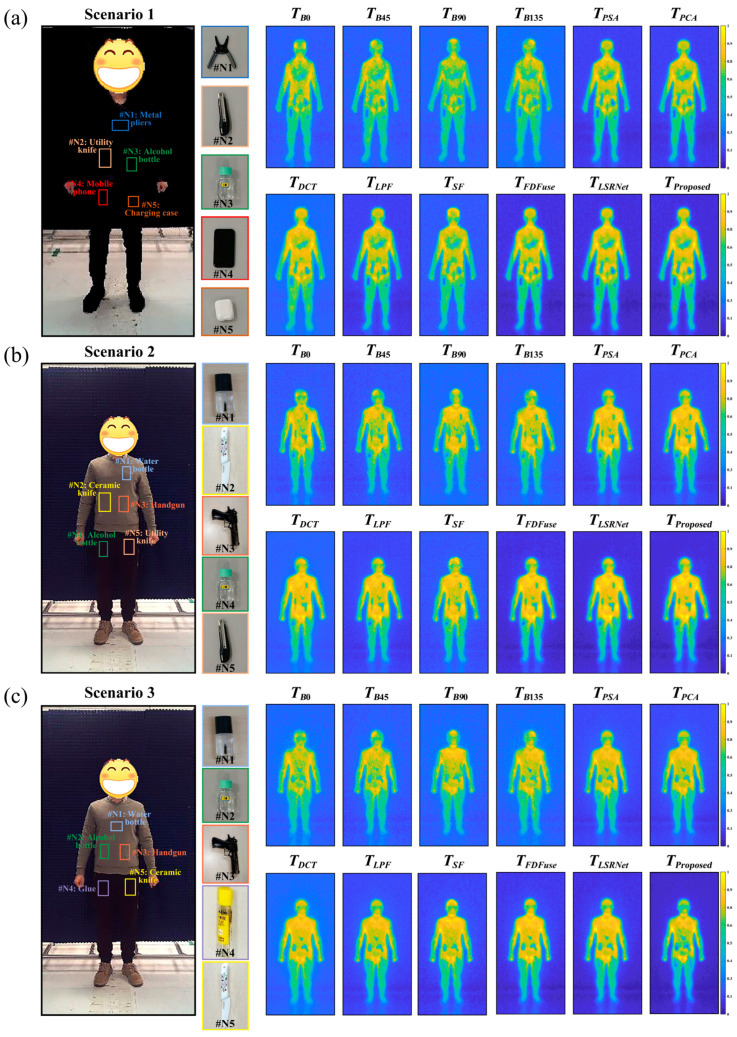
Imaging results for concealed object detection in the human body. (**a**) Detection imaging results for experimental scenario 1, with concealed objects including a metal pliers (#N1), a utility knife (#N2), an alcohol bottle (#N3), a mobile phone (#N4), and a charging case (#N5). (**b**) Detection imaging results for experimental scenario 2, with concealed objects including a water bottle (#N1), a ceramic knife (#N2), a handgun (#N3), an alcohol bottle (#N4), and a utility knife (#N5). (**c**) Detection imaging results for experimental scenario 3, with concealed objects including a water bottle (#N1), an alcohol bottle (#N2), a handgun (#N3), a glue (#N4), and a ceramic knife (#N5).

Two volunteers were recruited for the experiment, as illustrated in Figure 6. One volunteer participated in Scenario 1, while the other volunteer performed Scenarios 2 and 3. In each scenario, five types of concealed objects were randomly placed on the volunteer’s chest, abdomen, and thigh pockets, with all objects positioned inside the clothing and close to skin. Imaging was conducted using a W-band focal plane scanning system [37], operating within the 70–110 GHz frequency range (bandwidth: 40 GHz). The radiometer channels featured a noise figure better than 3.5 dB, with an integration time of 280 μs, achieving a radiometric sensitivity better than 0.5 K.

A high-density polyethylene dielectric lens with a diameter of 460 mm was employed to focus the millimeter waves, and the observation distance was 2.5 m. Imaging under different linear polarizations was realized by rotating the radiometer and feed antenna. Polarization channel calibration was performed using the two-point calibration method: a blackbody absorbing material (emissivity: 0.999) was placed in front of the system, with its physical temperature varying between 25 °C and 60 °C, while a cold temperature reference was obtained by immersing the material in liquid nitrogen (approximately 77 K). Calibration was conducted prior to each polarization angle acquisition to compensate for system drift. To assess stability, each volunteer/scenario was independently measured three times. All experiments were carried out indoors at an ambient temperature of approximately 25 °C, and linear polarization images at 0°, 45°, 90°, and 135° were acquired using the W-band PMMW system.

### 4.1. Concealed Contraband Detection Imaging Results

In this paper, we adopt an MSMG-PCNN model to fuse the FS and CS layers. For the PCNN, the decay time constant *α_θ_* is set to 3, and the amplification factors *V_L_* and *V_θ_* are 1 and 20, respectively. The number of iterations is fixed at 100, with the stopping rule defined as reaching the maximum iteration count. A 3 × 3 connection kernel is used as the neighborhood size. For the morphological gradient, the scale factor is *v* = 3, and the basic structural element *MS*_1_ is a 3 × 3 all-ones structuring element. The GF is applied with σ = 20 and a kernel size of 5 × 5. In the WACF decomposition, the number of iterations per scale is set to 2. For the EA fusion, the gain coefficient is *α_G_* = 4.

As shown in Figure 6, the results of three sets of concealed contraband detection imaging experiments are presented, corresponding to three different scenarios from top to bottom in sequence. Figure 6a shows the detection imaging results for Scenario 1, where five concealed objects are annotated with color boxes, including metal pliers (#N1), a utility knife (#N2), an alcohol bottle (#N3), a mobile phone (#N4), and a charging case (#N5). Figure 6b displays the image processing result for Scenario 2, containing a water bottle (#N1), a ceramic knife (#N2), a handgun (#N3), an alcohol bottle (#N4), and a utility knife (#N5). Figure 6c is the imaging result for Scenario 3, which includes a water bottle (#N1), an alcohol bottle (#N2), a handgun (#N3), a glue (#N4), and a ceramic knife (#N5).

As can be observed from the imaging results, all four linear polarization modes—horizontal (*T_B_*_0_), 45° (*T_B_*_45_), vertical (*T_B_*_90_), and 135° (*T_B_*_135_) linear polarization—struggle to effectively distinguish concealed contraband from the human body background, thereby significantly reducing the detection probability. This reveals an inherent limitation of linear polarization in PMMW security checks: its detection performance is highly dependent on the angle between the direction of the target and the polarization direction. For instance, a knife exhibits the strongest signal when aligned parallel to the polarization direction, while the response weakens considerably under orthogonal orientation, easily leading to missed detections. Additionally, non-metallic or structurally complex concealed contraband is difficult to identify due to its weak polarimetric signature. Moreover, the polarization wave scattering caused by the curvature and contour of the human body will also introduce interference and increase the difficulty of image interpretation.

To improve the detection capability of concealed objects, we further investigated multipolarization fusion methods. The results processed by seven fusion methods—polarization summation average (PSA) [25], principal component analysis (PCA) [38], discrete cosine transform (DCT) [39], laplacian pyramid fusion (LPF) [40], subregion fusion (SF) [25], and two advanced deep learning fusion models, FDFuse [41] and LSRNet [42]—along with the proposed fusion method are presented in Figure 6. It can be seen that the proposed fusion strategy effectively suppresses image noise while enhancing the intensity contrast between the concealed object and the human background, and presenting a more complete object shape and clearer contour features.

### 4.2. Performance Analysis

As can be observed from the detection imaging results in Figure 6, the proposed method effectively reconstructs the shape and contour features of concealed contraband. To further quantitatively evaluate its reconstruction performance, we introduce entropy [43], blind/referenceless image spatial quality evaluator (BRISQUE) [44], natural image quality evaluator (NIQE) [45], perception-based image quality evaluator (PIQE) [46], and signal-to-noise ratio (SNR) [25] for comprehensive analysis.

In PMMW imaging, the acquired images inherently suffer from SNR and pronounced noise interference. Under such conditions, high entropy values often originate not from meaningful target information but from background noise and random fluctuations. Pursuing high entropy alone may instead indicate insufficient noise suppression, which is detrimental to subsequent detection and recognition tasks. The core of the proposed method lies in enhancing the brightness temperature contrast of concealed targets and emphasizing target contours and structural information, rather than preserving all textural details including noise. Consequently, in the fused image, fluctuations in background regions are effectively suppressed, target information is strengthened, and the pixel distribution becomes more concentrated. The moderate reduction in entropy therefore reflects the effectiveness of the method, rather than signifying information loss. In fact, in PMMW tasks, lower entropy generally corresponds to a clearer background and more salient targets. Hence, entropy should not be interpreted in isolation as “information richness”. Instead, it must be jointly interpreted with task-relevant metrics such as SNR and detection accuracy.

On the other hand, although no-reference image quality assessment metrics such as BRISQUE, NIQE, and PIQE were originally designed for natural images, their core function is to measure structural integrity, perceptual quality, and the degree of distortion, and they are not strictly confined to natural scenes. These metrics are built upon spatial-domain statistical features, patch-based statistical models, or perceptual features, and they exhibit high sensitivity to blur, noise, block artifacts, and structural distortions—precisely the critical factors that need to be evaluated in PMMW image fusion. In PMMW imaging, quality assessment focuses on edge preservation, structural clarity, and noise suppression, which align closely with the statistical characteristics captured by the aforementioned no-reference metrics. Therefore, employing these metrics to evaluate the fusion performance of the proposed method is both reasonable and valid.

For the three experimental scenarios depicted in Figure 6, the computed results of each evaluation metric (entropy, BRISQUE, NIQE, PIQE) are summarized in Table 1. The data demonstrate that the proposed fusion strategy outperforms all comparative methods across all metrics, achieving the best PMMW imaging reconstruction performance. Furthermore, as evidenced by the SNR value for each concealed contraband in Table 2, the proposed method achieves the highest SNR value. This significantly enhances the contrast between concealed contraband and the surrounding background, thereby effectively improving target detectability.

### 4.3. Performance on ROC Curves

To comprehensively assess the detection performance, this paper adopts the ROC curve proposed in [47] as an analytical tool. This curve clearly illustrates the trade-off between detection sensitivity and specificity by plotting the relationship between the true positive rate (TPR) and false positive rate (FPR) across varying thresholds. The area under the curve (AUC) serves as a measure of the overall performance, where TPR reflects the detection sensitivity and FPR is related to its specificity. Their respective formulas are expressed as follows:

(47) $$\mathrm{TPR}=\frac{\mathrm{TP}}{\mathrm{TP}+\mathrm{FN}}$$

(48) $$\mathrm{FPR}=\frac{\mathrm{FP}}{\mathrm{FP}+\mathrm{TN}}$$

where TP (true positive) denotes pixels that truly belong to the target object and are correctly identified as such. TN (true negative) refers to pixels that truly belong to the background and are correctly classified as background. FP (false positive) represents pixels that truly belong to the background but are incorrectly identified as the target object. FN (false negative) indicates pixels that truly belong to the target object but are incorrectly identified as background.

Regarding the generation of ROC curves, we need to further clarify the following two points. First, we provide a detailed description of the threshold setting protocol: after normalizing the pixel values of the fused images to the range [0, 1], we traverse all possible thresholds at a step size of 0.01, and calculate the TPR and FPR at each step, thus generating a complete ROC curve. Second, regarding the generation of ground-truth masks, three researchers independently performed manual annotation based on the actual positions and contours of concealed objects in the original PMMW images. The final binary masks were determined by majority voting, while background regions were selected from typical areas free of interference around the targets. All methods under comparison (including the proposed method and the seven competing methods) strictly use the identical masks for pixel-wise evaluation, ensuring a consistent and fair basis for computing TP, FP, TN, and FN.

**Figure 7:**
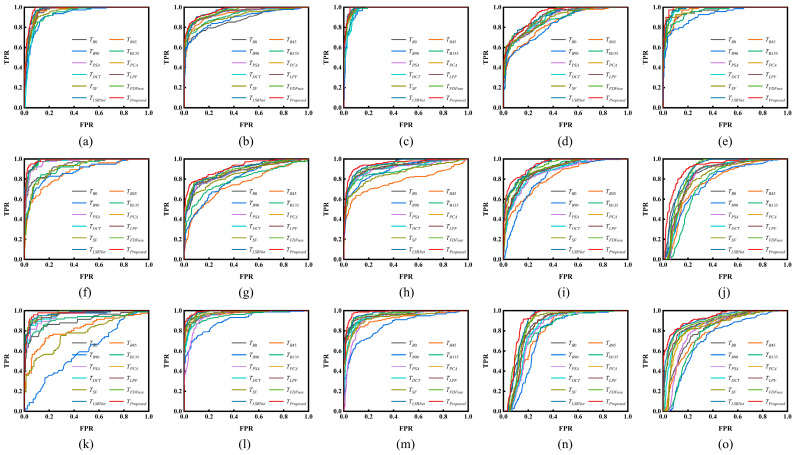
Comparison of ROC curves for multiple methods in each concealed object. (**a**–**e**) Concealed object #N1~#N5 of experimental scenario 1 results. (**f**–**j**) Concealed object #N1~#N5 of experimental scenario 2 results. (**k**–**o**) Concealed object #N1~#N5 of experimental scenario 3 results.

As illustrated in Figure 7, the ROC curves provide a visual representation of the detection outcomes for each concealed object across the three experimental scenarios. The experimental results demonstrate that the ROC curve of the proposed method is consistently closest to the upper-left corner in all detection tasks and achieves the largest AUC values. This finding indicates that the proposed method maintains a significant advantage in detecting concealed objects, exhibiting superior discriminative ability and higher detection accuracy. In other words, the proposed method attains the largest AUC for concealed contraband in all cases, further confirming its excellent detection performance.

### 4.4. Ablation Experiments

To further validate the contribution of each key component in the proposed framework, we conducted a series of ablation experiments on the first experimental scenario. Specifically, we evaluated the following variants: (1) Decomposition architecture: replacing GWACF with Gaussian filtering alone or WACF alone; (2) Texture layer fusion: removing the MSMG modulation from PCNN; (3) Background layer fusion: replacing EA fusion with simple weighted averaging; (4) Decomposition layers: using one-layer (BS only) or two-layer (BS + FS) decomposition; and (5) Input construction: using the original four-channel polarization images instead of TA/TB. The quantitative results are shown in Table 3, Table 4, Table 5, Table 6 and Table 7. The complete model consistently outperformed all variants across all evaluation metrics, confirming the necessity and effectiveness of each proposed module. In particular, GWACF achieved the best edge-preserving decomposition, MSMG-PCNN enhanced texture fusion quality, EA fusion maintained structural integrity, and the three-level decomposition provided the optimal multi-scale representation.

### 4.5. Time Complexity Analysis

The proposed method mainly consists of three parts: GWACF multi-scale decomposition, MSMG-PCNN fusion (applied to the FS and CS layers), and energy attribute fusion (applied to the BS layer). Let the size of a single input image be *H* × *W*. The number of GWACF decomposition scales is set to *S* = 3, and during the WACF decomposition process, the number of iterations at each scale is set to *L*. The number of MSMG scales is denoted as *R*. The number of PCNN iterations is *N*, and the size of the neighborhood window is *K* × *K*.

(1) GWACF decomposition: Each layer consists of GF operation (complexity *O*(*HW*)) and WACF operation. WACF involves convolution with eight directional kernels, and performing *L* iterations for each convolution yields a complexity of *O*(*K*^2^*LHW*). Therefore, the complexity of a single layer is *O*(8*K*^2^*LHW*). Consequently, the total complexity of the three-layer GWACF decomposition is *O*(3·(*HW* + 8*K*^2^*LHW*)), which can be further expressed as *O*(*HW* + *K*^2^*LHW*).
(2) MSMG-PCNN fusion: This is used for the FS and CS layers (a total of 2*S* = 6 layers). For each scale *v* in MSMG, morphological dilation and erosion operations are performed on the image. The morphological operation for each pixel requires traversing all pixels within the window, so the complexity for a single scale is O(*HWv*^2^). Summing over all scales yields *O*(*HWR*^3^). In the PCNN, each iteration involves computing the neighborhood connection weights (complexity *O*(*K*^2^*HW*)) and updating the internal activity (complexity *O*(*HW*)). With a total of *N* iterations, the complexity for a single layer is *O*(*NK*^2^*HW*). Consequently, the overall complexity of MSMG-PCNN fusion is *O*(6·(*HWR*^3^ + *NK*^2^*HW*)), which can be further simplified to *O*(*HWR*^3^ + *NK*^2^*HW*).
(3) Energy attribute fusion (BS layer): This involves only pixel-wise mean and exponential operations, yielding a complexity of *O*(*HW*).

In summary, the overall complexity of the proposed method is:

O(*HW*·(*R*^3^ + *NK*^2^ + *K*^2^*L*))

where *R* = *K* = 3, *L* = 2, and *N* is set to 100, so the overall complexity is linearly related to the size of the image.

To verify the practical efficiency, we measured the average running time of each algorithm on the same hardware platform (a computer equipped with an i7-12700H CPU and a GeForce RTX 3060 GPU). The experimental results are presented in Table 8. For images with a resolution of 175 × 360, the average processing time of the proposed method is approximately 0.69 s. Although this time is higher than those of the other fusion methods, considering the significant improvement in detection accuracy and edge preservation achieved by our method, this computational cost remains within an acceptable range for practical security inspection scenarios. It is anticipated that the use of higher-performance processors, combined with further exploration of acceleration algorithms, could potentially enable real-time imaging at video rates in the future.

## 5. Conclusions

This paper addresses the insufficient application of multi-polarization technology in PMMW security imaging by proposing a multi-scale edge-preserving image decomposition framework integrated with a gradient-domain PCNN fusion strategy. The method employs a GWACF model to decompose images into three layers—FS, BS, and CS layers—which are subsequently fused using gradient-domain PCNN and energy-attribute methods. This enables effective complementary of multi-polarization information while preserving essential texture and edge details. Experimental results demonstrate that the proposed fusion method outperforms existing mainstream methods across multiple evaluation metrics, significantly enhancing target contour and structural information while providing superior image quality for subsequent detection and segmentation tasks. This study has verified the potential of the multi-polarization PMMW technology in the detection of concealed objects in the human body, and it is applicable to scenarios such as non-intrusive security checks at smart transportation hubs, covert security protection in public places, and non-destructive flaw detection in industries.

In terms of future research works, the following two aspects will be focused on: firstly, further optimization of the fusion algorithm to improve computational efficiency and achieve real-time imaging capability at video rate, and secondly, exploration of integration with deep learning-based fusion methods to enhance the extraction and characterization of multi-polarization target features.

## Figures and Tables

**Table 1:** Computed results of evaluation metrics for various processing methods. The bold values indicate the best performance.

Scenario	Method	Entropy	BRISQUE	NIQE	PIQE
Scenario 1	*T_B_*_0_	6.91	40.58	12.87	54.41
	*T_B_*_45_	6.89	39.25	13.88	56.50
	*T_B_*_90_	6.86	38.43	15.40	55.47
	*T_B_*_135_	6.83	38.07	13.90	55.06
	*T_PSA_*	6.75	33.94	11.53	42.80
	*T_PCA_*	6.69	34.06	10.08	42.03
	*T_DCT_*	6.54	35.73	10.22	42.66
	*T_LPF_*	6.71	34.46	10.14	41.46
	*T_SF_*	6.82	38.29	13.91	53.40
	*T_FDFuse_*	6.21	34.58	10.31	42.65
	*T_LSRNet_*	6.03	33.31	10.18	40.46
	*T_Proposed_*	**5.91**	**29.50**	**10.03**	**32.15**
Scenario 2	*T_B_*_0_	6.86	41.09	12.92	55.19
	*T_B_*_45_	6.85	41.10	11.31	56.74
	*T_B_*_90_	6.74	40.05	12.29	54.38
	*T_B_*_135_	6.87	41.02	11.85	55.09
	*T_PSA_*	6.60	35.91	10.45	45.45
	*T_PCA_*	6.11	42.49	14.79	63.39
	*T_DCT_*	6.49	36.17	10.40	44.13
	*T_LPF_*	6.38	33.44	10.45	43.19
	*T_SF_*	6.84	40.25	11.96	56.57
	*T_FDFuse_*	5.46	33.48	8.87	43.29
	*T_LSRNet_*	5.40	31.63	8.06	41.47
	*T_Proposed_*	**5.25**	**21.51**	**6.01**	**23.09**
Scenario 3	*T_B_*_0_	6.79	40.96	12.43	55.79
	*T_B_*_45_	6.85	41.08	11.77	56.56
	*T_B_*_90_	6.83	41.36	12.67	57.74
	*T_B_*_135_	6.82	40.98	12.62	55.57
	*T_PSA_*	6.61	36.48	10.82	45.40
	*T_PCA_*	6.63	36.59	10.70	45.61
	*T_DCT_*	6.50	40.63	10.56	44.66
	*T_LPF_*	6.60	35.95	10.13	45.40
	*T_SF_*	6.59	26.01	7.37	28.05
	*T_FDFuse_*	6.21	35.66	5.13	28.43
	*T_LSRNet_*	6.17	32.19	4.79	25.19
	*T_Proposed_*	**6.03**	**18.77**	**4.24**	**18.44**

**Table 2:** SNR (Unit: dB) for each concealed object in the experimental results. The bold values indicate the best performance.

Scenario	Number	Concealed Object	*T_B_*_0_	*T_B_*_45_	*T_B_*_90_	*T_B_*_135_	*T_PSA_*	*T_PCA_*	*T_DCT_*	*T_LPF_*	*T_SF_*	*T_FDFuse_*	*T_LSRNet_*	*T_Proposed_*
Scenario 1	#N1	Metal pliers	6.89	7.15	5.72	6.01	7.67	7.84	7.69	7.98	7.18	8.08	8.19	**9.39**
	#N2	Utility knife	10.01	10.71	8.45	10.43	11.79	11.78	11.66	12.16	11.51	12.15	12.33	**13.56**
	#N3	Alcohol bottle	11.40	11.35	10.19	10.81	11.76	12.08	12.06	12.27	11.54	12.71	12.94	**13.61**
	#N4	Mobile phone	9.13	9.46	7.16	10.05	10.42	10.49	10.46	10.65	10.28	10.86	11.02	**11.35**
	#N5	Charging case	10.72	10.42	8.72	9.58	11.39	12.46	12.41	12.55	11.67	12.04	12.26	**13.03**
Scenario 2	#N1	Water bottle	9.02	6.47	7.40	7.22	11.29	11.27	11.31	11.41	6.88	11.89	12.01	**12.92**
	#N2	Ceramic knife	6.29	1.71	2.79	3.98	7.51	7.55	7.52	7.53	6.47	8.06	8.37	**9.29**
	#N3	Handgun	9.66	6.29	8.31	9.23	10.91	10.88	10.92	11.16	7.95	11.37	11.53	**11.72**
	#N4	Alcohol bottle	7.08	3.35	1.84	7.57	6.15	6.22	6.16	6.17	6.72	7.29	7.51	**9.48**
	#N5	Utility knife	6.19	5.63	3.73	5.82	6.81	6.86	6.84	6.87	6.23	7.93	8.26	**8.95**
Scenario 3	#N1	Water bottle	10.21	6.54	1.08	11.96	12.41	12.39	12.45	12.61	3.66	13.62	13.90	**14.77**
	#N2	Alcohol bottle	9.75	11.24	8.71	10.57	12.13	12.15	12.18	12.77	12.55	13.15	13.49	**15.73**
	#N3	Handgun	10.17	10.49	7.02	10.03	11.29	11.31	11.05	11.34	10.99	11.92	12.17	**13.24**
	#N4	Glue	5.35	2.81	2.05	3.65	4.64	4.63	4.83	5.03	5.77	6.85	7.08	**8.31**
	#N5	Ceramic knife	2.15	1.32	0.86	1.73	2.68	2.71	2.76	3.54	3.89	4.57	4.65	**6.87**

**Table 3:** SNR (Unit: dB) for each concealed object in the decomposition architecture ablation experiment results. The bold values indicate the best performance.

	Metal Pliers	Utility Knife	Alcohol Bottle	Mobile Phone	Charging Case
GF only	6.86	10.98	9.45	8.12	10.62
WACF only	7.31	11.67	11.38	9.71	11.70
GWACF (complete)	**9.39**	**13.56**	**13.61**	**11.35**	**13.03**

**Table 4:** SNR (Unit: dB) for each concealed object in the texture layer fusion ablation experiment results. The bold values indicate the best performance.

	Metal Pliers	Utility Knife	Alcohol Bottle	Mobile Phone	Charging Case
PCNN (without MSMG)	8.23	11.92	12.78	10.16	11.57
PCNN + MSMG (complete)	**9.39**	**13.56**	**13.61**	**11.35**	**13.03**

**Table 5:** SNR (Unit: dB) for each concealed object in the background layer fusion ablation experiment results. The bold values indicate the best performance.

	Metal Pliers	Utility Knife	Alcohol Bottle	Mobile Phone	Charging Case
Simple weighted average	8.77	12.81	12.93	10.82	12.35
EA fusion (complete)	**9.39**	**13.56**	**13.61**	**11.35**	**13.03**

**Table 6:** SNR (Unit: dB) for each concealed object in the different decomposition layers ablation experiment results. The bold values indicate the best performance.

	Metal Pliers	Utility Knife	Alcohol Bottle	Mobile Phone	Charging Case
BS only	5.41	8.85	8.08	6.93	7.74
BS+FS	7.53	10.75	10.66	8.51	9.18
BS + FS + CS (complete)	**9.39**	**13.56**	**13.61**	**11.35**	**13.03**

**Table 7:** SNR (Unit: dB) for each concealed object in the input construction method ablation experiment results. The bold values indicate the best performance.

	Metal Pliers	Utility Knife	Alcohol Bottle	Mobile Phone	Charging Case
*T_B_*_0_, *T_B_*_45_, *T_B_*_90_, *T_B_*_135_ direct fusion	8.88	12.49	12.73	10.17	11.36
*T_A_*/*T_B_* construction (complete)	**9.39**	**13.56**	**13.61**	**11.35**	**13.03**

**Table 8:** Average execution time of various methods (Unit: s).

Method	Average Execution Time
*T_PSA_*	0.0085
*T_PCA_*	0.2686
*T_DCT_*	0.0153
*T_LPF_*	0.3631
*T_SF_*	0.3327
*T_FDFuse_*	0.0968
*T_LSRNet_*	0.1501
*T_Proposed_*	0.6873

## Data Availability

All data relevant to this study are available from the corresponding author upon reasonable request.

## List of Acronyms

The following abbreviations are used in this manuscript:

AoP	Angle of polarization
AUC	Area under the curve
BRISQUE	Blind/referenceless image spatial quality evaluator
BS	Base structural
BT	Brightness temperature
CS	Coarse structural
DCT	Discrete cosine transform
DoLP	Degree of linear polarization
EA	Energy attribute
FN	False negative
FP	False positive
FPR	False positive rate
FS	Fine structural
GWACF	Gaussian and weighted average curvature filtering
LPF	Laplacian pyramid fusion
LPR	Linear polarization ratio
MSMG	Multi-scale morphological gradient
NIQE	Natural image quality evaluator
PCA	Principal component analysis
PCNN	Pulse-coupled neural network
PDoP	Passive degree of polarization
PIQE	Perception based image quality evaluator
PMMW	Passive millimeter-wave
PSA	Polarization summation average
SF	Subregion fusion
SNR	Signal-to-noise ratio
TN	True negative
TP	True positive
TPR	True positive rate
WACF	Weighted average curvature filtering
